# *Momordica charantia* Extract Ameliorates Melanoma Cell Proliferation and Invasion into Mouse Lungs by Suppressing PAX3 Expression

**DOI:** 10.3390/ijms252312800

**Published:** 2024-11-28

**Authors:** Keiichi Hiramoto, Hirotaka Oikawa

**Affiliations:** Department of Pharmaceutical Sciences, Suzuka University of Medical Science, 3500-3 Minamitamagaki, Suzuka 513-8607, Japan; oikawah@suzuka-u.ac.jp

**Keywords:** *Momordica charantia* extract, PAX3, PTEM, Akt, MITF

## Abstract

Melanomas, which develop on malignant transformations of melanocytes, are highly malignant and prone to metastasis; therefore, effective drugs are required. The *Momordica charantia* (MC) extract has been shown to suppress cancer cell proliferation and invasion; however, the effect of the MC extract on melanoma in living organisms remains unclear. In this study, we investigated the mechanism underlying the amelioration of melanoma cell extravasation into mouse lungs by the MC extract. Male C57BL/6j mice (aged 8 weeks) were injected with B16 melanoma cells (1 × 10^5^ cells/mouse). Subsequently, they were orally administered the MC extract daily for 2 weeks; mouse lung samples were obtained on the final day and analyzed. The MC extract ameliorated melanoma proliferation and infiltration into the lungs caused by melanoma cell treatment. It also increased phosphatase and tensin homolog deletion from chromosome 10 and suppressed paired box gene 3 (PAX3) and the phosphatidylinositol trisphosphate/RAC-alpha serine/threonine-protein kinase/mammalian target of rapamycin complex 1 signaling. Furthermore, it decreased microphthalmia-associated transcription factors and induced the suppression of cyclin-dependent kinase 2, hepatocyte growth factor receptor, B-cell/CLL lymphoma 2, and Ras-related proteins. Our findings suggest that the MC extract suppresses tumor survival genes by regulating PAX3, thereby ameliorating melanoma proliferation and invasion.

## 1. Introduction

Malignant melanoma is a malignant tumor derived from melanocytes. Although malignant melanoma is not a common skin cancer, it accounts for 79% of all skin cancer-related deaths. It is a malignant tumor that spreads from a small primary tumor via the blood and lymphocytes and metastasizes to various sites [1,2]. Tumor malignancy is attributable to frequent metastasis and a poor response to chemotherapy and radiation therapy.

Genetic [3,4,5] and environmental [6] factors are intricately involved in malignant melanoma development. The most common genetic abnormality in Japanese patients with melanoma occurs in the v-Raf murine sarcoma viral oncogene homolog B (BRAF) gene [7] (~30% of Japanese patients with melanoma); abnormalities are also observed in the KIT proto-oncogene, receptor tyrosine kinase (KIT) gene [7], and neuroblastoma RAS viral (v-ras) oncogene homolog (NRAS) gene [8]. Melanocytes are present in the basal layer of the epidermis, dermis, and hair matrix in the skin; they synthesize and supply melanin pigment to the surrounding epidermal cells via melanosomes. In 75% of cases, melanomas arise de novo from melanocytes in normal skin, while in 25% of cases, they arise from the malignant transformation of existing benign melanocyte lesions. In addition, intracellular signal transduction pathways, such as the mitogen-activated protein kinase (MAPK) pathway [9,10] and the phosphoinositide 3-kinase (PI3K) pathway [11], play important roles in melanoma cell proliferation.

Melanomas develop in various tissues and are mainly surgically excised during early developmental stages. Treatment approaches using a combination of therapies such as chemotherapy, immunotherapy, and radiation therapy are used as the disease progresses. Recently, antibody drugs targeting immune cells responding to melanomas have also been used. However, in many cases, the melanoma is highly malignant, leading to limited treatment effectiveness, and antibody drugs are expensive, which greatly increases treatment costs.

Bitter melon (*Momordica charantia* [MC]) is a creeping annual plant from the Cucurbitaceae family. MC has long been studied worldwide for its antidiabetic effects. An aqueous MC extract was found to promote insulin secretion from β cells in obese hyperglycemic mice [12] and also inhibit pancreatic peroxidation and reduce β-cell apoptosis in streptozotocin-induced hyperglycemic mice [13]. Recently, bitter melon components have been shown to promote the secretion of incretin, a gastrointestinal hormone that promotes pancreatic insulin secretion, and to inhibit the activity of dipeptidyl peptidase-4, an incretin-inactivating enzyme [14,15]. Bitter melon water extract has been reported to reduce triglyceride and very low-density lipoprotein (VLDL) levels in rats with diabetes, ameliorate lipid metabolism abnormalities, and normalize liver and kidney functions [16,17].

Bitter melon extract has also been found to have antitumor effects. The extract induces apoptosis in human leukemia (HL60) and B16 melanoma cells [18,19]; these findings suggest that the extract exerts a cancer-suppressive effect by inducing cancer cell apoptosis. Furthermore, it specifically induces the apoptosis of cancer cells but not normal cells [19,20]. The extract was found to induce the inhibition of cancer metastasis in vivo and in vitro in rat prostate cancer cells [21].

Thus, although many researchers have reported the antitumor effects of bitter melon extract, its effect on melanoma in vivo remains unclear. Therefore, we aimed to investigate the effect of the MC extract on melanoma cell invasion and proliferation by intravenously injecting B16 melanoma cells into mice. Furthermore, we investigated the mechanism underlying the effect of the MC extract on melanoma.

## 2. Results

### 2.1. Effects of MC Extract on Melanoma Cell Infiltration and Proliferation

**Figure 1:**
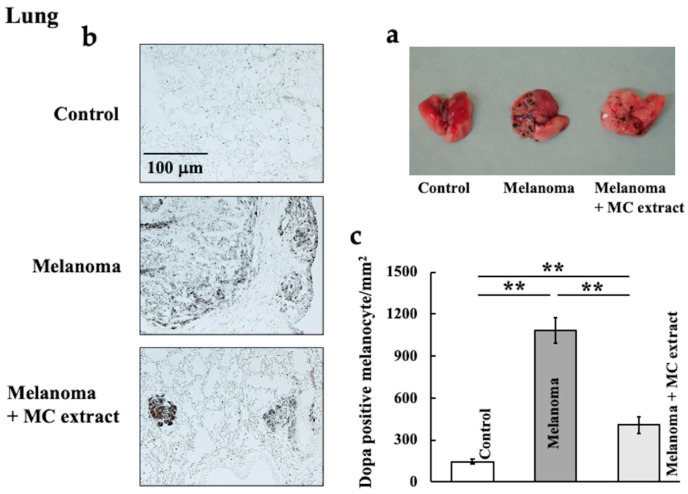
Effects of *Momordica charantia* (MC) extract treatment on melanoma cell infiltration and proliferation in mouse lungs. At 2 weeks after study initiation, the lungs were macroscopically analyzed (**a**); the number of DOPA-positive cells was measured by DOPA staining (**b**,**c**). Values are expressed in terms of mean ± SD (*n* = 5 animals). ** *p* < 0.01. Scale bar = 100 μm.

At 2 weeks after intravenous melanoma cell injection, melanoma cell infiltration and proliferation in mouse lungs were macroscopically and microscopically analyzed (Figure 1). Macroscopic analysis showed that melanoma cell infiltration and proliferation had significantly increased; however, MC extract administration had reduced the counts of these cells (Figure 1a). Additionally, tyrosine induced melanin production in DOPA-positive cells. In malignant melanoma, the proportion of dopa-positive cells increases; therefore, these cells function as a disease parameter [22]. Dopa-positive melanocytes also significantly increased in mice injected with melanoma cells and decreased in mice administered the MC extract (Figure 1b,c).

### 2.2. Effect of MC Extract on Paired Box Gene 3 (PAX3) Expression in Mouse Lungs

**Figure 2:**
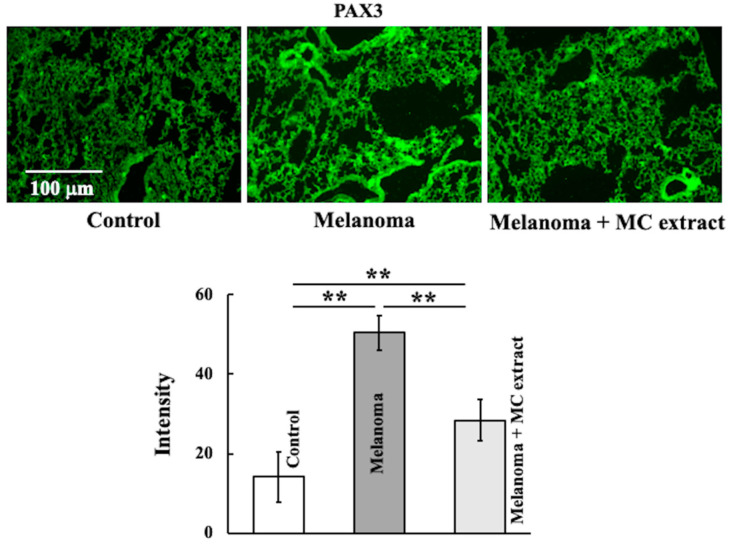
Effects of MC extract treatment on paired box gene 3 (PAX3) expression in the lungs of melanoma-bearing mice. At 2 weeks after study initiation, PAX3 expression was observed. Values are expressed in terms of mean ± SD (*n* = 5 animals). Intensity was calculated from five random visual fields with a constant area by using the ImageJ software. ** *p* < 0.01. Scale bar = 100 µm.

We investigated PAX3 expression in the lungs of mice injected with B16 mouse melanoma cells (Figure 2). PAX3 expression increased upon melanoma cell injection and decreased upon MC extract administration.

### 2.3. Effect of MC Extract on Phosphatase and Tensin Homolog Deletion from Chromosome 10 (PTEN), PI3K, and Phosphatidylinositol Trisphosphate (PIP3) Expression Levels in Mouse Lungs

**Figure 3:**
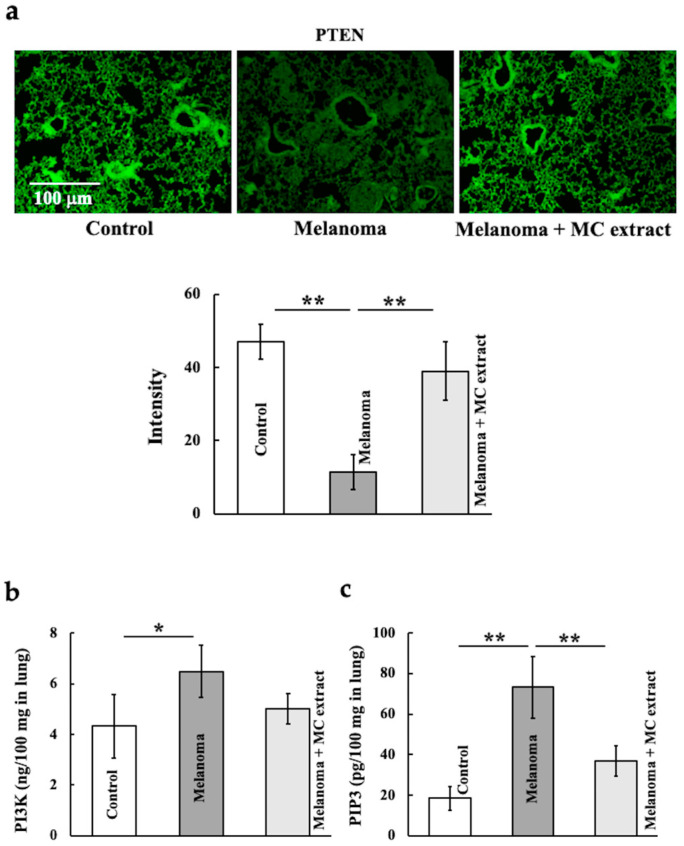
Effects of MC extract treatment on phosphatase and tensin homolog deletion from chromosome 10 (PTEN), phosphoinositide 3-kinase (PI3K), and phosphatidylinositol trisphosphate (PIP3) levels in the lungs of melanoma-bearing mice. The PTEN (**a**), PI3K (**b**), and PIP3 (**c**) levels were analyzed. Values are expressed in terms of mean ± SD (*n* = 5 animals). Intensity was calculated from five random visual fields with a constant area by using the ImageJ software. PI3K and PIP3 were measured using enzyme-linked immunosorbent assay (ELISA) kits. * *p* < 0.05; ** *p* < 0.01. Scale bar = 100 μm.

We investigated the levels of PTEN, PI3K, and PIP3, which are affected by PAX3. PTEN expression decreased upon melanoma cell injection but increased upon MC extract administration and remained unchanged in the control group (Figure 3a). PI3K and PIP3 levels increased upon melanoma cell injection (Figure 3b,c). The PIP3 level decreased upon MC extract administration and remained unchanged from that in the control group.

### 2.4. Effect of MC Extract on RAC-Alpha Serine/Threonine-Protein Kinase (Akt), mTORC, Ribosomal Protein S6 (rpS6), Ribosomal Protein S6 Kinase Beta-1 (S6K1), Ki67, and Cyclin D Levels in the Skeletal Muscle

**Figure 4:**
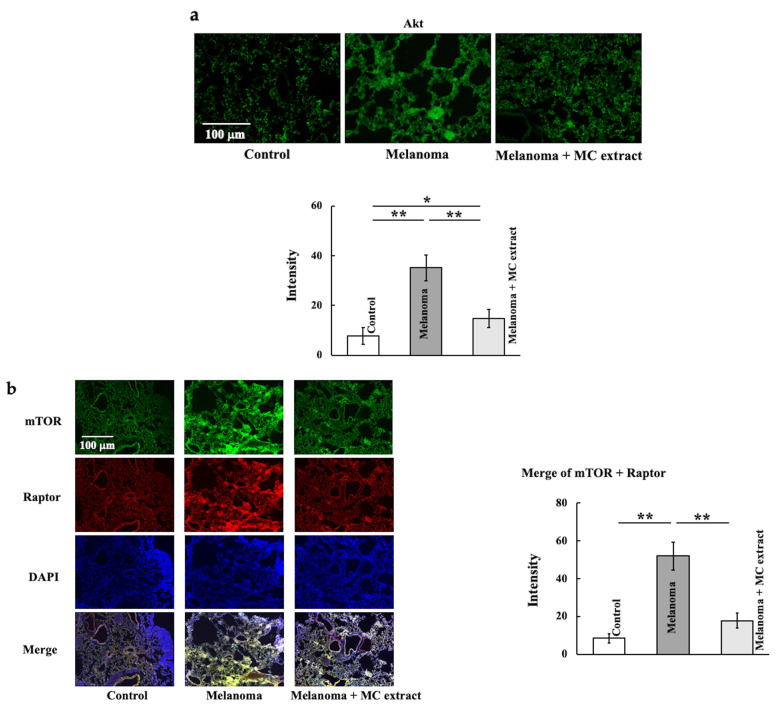

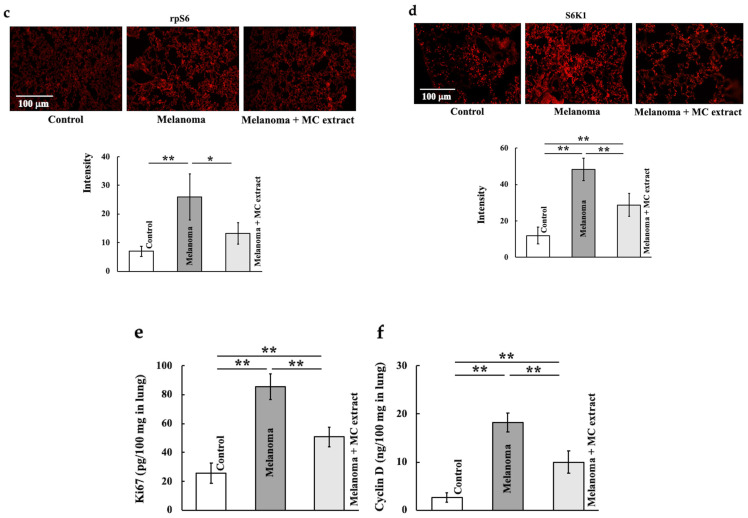
Effects of MC extract treatment on the signal transduction from RAC-alpha serine/threonine-protein kinase (Akt) to protein synthesis in the lungs of melanoma-bearing mice. The Akt (**a**), mTORC1 (**b**), rpS6 (**c**), S6K1 (**d**), Ki67 (**e**), and cyclin D (**f**) levels were analyzed. Values are expressed in terms of mean ± SD (*n* = 5 animals). Intensity was calculated from five random visual fields with a constant area by using the ImageJ software. Ki67 and cyclin D were measured using ELISA kits. Scale bar = 100 μm. * *p* < 0.05; ** *p* < 0.01.

Akt (Figure 4a), mTORC1 (Figure 4b), rpS6 (Figure 4c), S6K1 (Figure 4d), Ki67 (Figure 4e), and cyclin D (Figure 4f) levels were also examined. mTORC1 expression was estimated from the merged areas of double staining for the mammalian target of rapamycin (mTOR) and regulatory-associated protein of mTOR (raptor). Akt, mTORC1, rpS6, S6K1, Ki67, and cyclin D levels significantly increased upon melanoma cell treatment and decreased upon MC extract administration.

### 2.5. Effect of MC Extract on Microphthalmia-Associated Transcription Factor (MITF), Cyclin-Dependent Kinase 2 (CDK2), Hepatocyte Growth Factor Receptor (C-Met), B-Cell/CLL Lymphoma 2 (Bcl2), and Ras-Related Protein (RAB27A) Expression in Mouse Lungs

**Figure 5:**
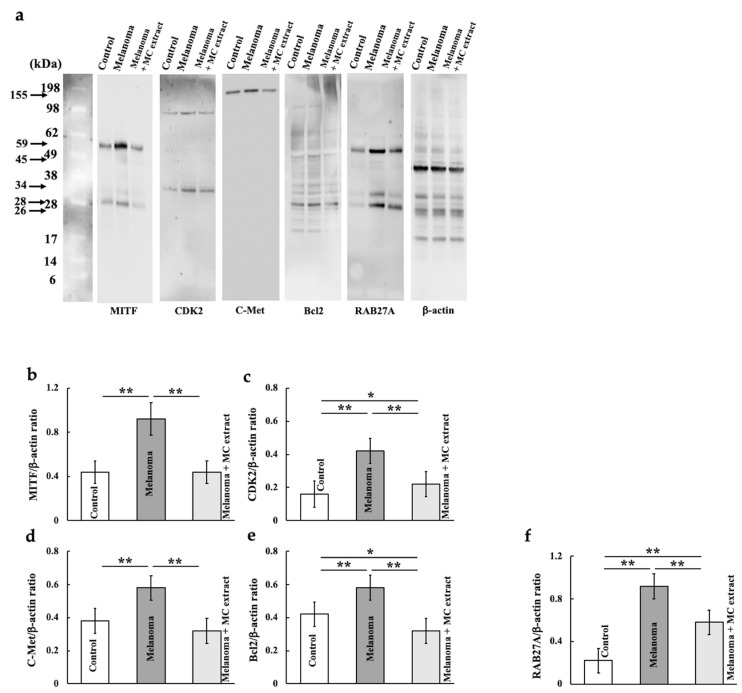
Effects of MC extract treatment on the expression levels of malignant tumor survival genes located downstream of microphthalmia-associated transcription factor (MITF) in the lungs of melanoma-bearing mice. The MITF (**a**,**b**), cyclin-dependent kinase 2 (CDK2) (**a**,**c**), hepatocyte growth factor receptor c-Met (**a**,**d**), B-cell/CLL lymphoma 2 (Bcl2) (**a**,**e**), and Ras-related protein (RAB27A) (**a**,**f**) levels were analyzed. Values are expressed in terms of mean ± SD (*n* = 5 animals). Intensity was calculated from five random visual fields with a constant area by using the ImageJ software. Scale bar = 100 μm. * *p* < 0.05; ** *p* < 0.01.

We investigated other genes regulated by PAX3, that is, MITF (Figure 5a,b), CDK2 (Figure 5a,c), c-Met (Figure 5a,d), Bcl2 (Figure 5a,e), and RAB27A (Figure 5a,f). The expression of these genes increased upon melanoma cell treatment and decreased upon MC extract administration.

## 3. Discussion

In this study, we found that MC extract administration reduced melanoma cell proliferation and invasion in mice injected with melanoma cells. The MC extract increased PAX3 expression, which had increased with melanoma cell treatment. Furthermore, the MC extract induced an increase in PTEN and decrease in PIK3, PIP3, Akt, mTOR, rpS6, and S6K1 levels. The MC extract also decreased the expression of MITF, CDK2, c-Met, Bcl2, and RAB27A, which had increased with melanoma cell treatment.

PI3K is activated by various stimuli such as cell growth factors, cytokines, chemokines, and hormones; activated PI3K phosphorylates PIP2 at position 3 to produce PIP3 [23]. PIP3 acts as a second messenger that activates intracellular signal transduction pathways including AktT. The PI3K/Akt/mTOR pathway is extremely important for tumorigenesis [24]. In the current study, MC extract administration also decreased the activity of PI3K, which had increased upon melanoma cell treatment. PTEN, which is also a tumor suppressor, decreases PI3K activation; it inhibits Akt by suppressing PI3K-mediated metabolic activity [25,26]. In the current study, MC extract administration induced an increase in PTEN expression, which had decreased upon melanoma cell treatment. This increase in PTEN expression may suppress PI3K/Akt/mTOR pathway activation. mTOR, a downstream effector of the PI3K/Akt signal transduction pathway, forms a complex. This study showed that mTORC1 levels increased because of mTOR and raptor coexpression [27]. mTORC1 regulates cell growth and proliferation by phosphorylating the translation regulators 4E-BP1 and S6K and regulates tumor cell-specific processes [28]. These findings suggest that the PI3K/Akt/mTORC1 pathway is activated and signals are transmitted from rpS6 to S6K1, thereby inducing tumor cell proliferation. Furthermore, previous research suggests that the MC extract suppresses this series of pathways by controlling PTEN and ameliorating melanoma cell proliferation and invasion.

In this study, alterations were identified in PAX3, which regulates the tumor suppressor protein PTEN, an essential negative regulator of the PI3K/Akt signaling pathway; PTEN regulates the resistance to cell proliferation [29]. This effect is attributable to the interaction of PAX3 with a putative homeodomain-binding motif within the PTEN promoter and the regulation of PTEN expression patterns by PAX3 [30]. Previous research suggests that the MC extract suppresses melanoma proliferation and invasion by decreasing PAX3 expression and regulating PTEN/PI3K/Akt signaling.

**Figure 6:**
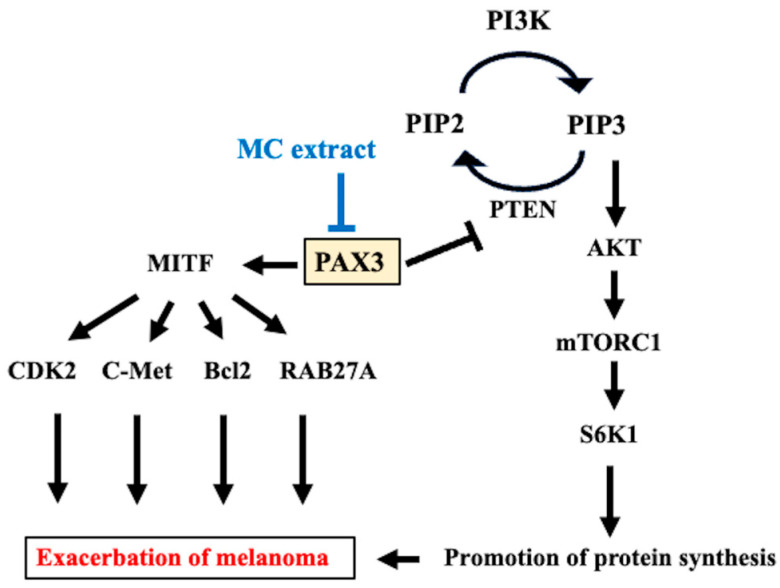
Mechanism underlying the inhibition of melanoma cell proliferation and invasion by the MC extract. PAX3 increases the activity of MITF and promotes the proliferation and invasion of melanoma by increasing CDK2, c-MET, Bcl2, and RAB27A. PAX3 also suppresses PTEN and activates signaling from PIP3 to AKT, mTORC1, and S6K1, which promotes melanoma proliferation. The MC extract improves melanoma by suppressing the MITF pathway and PIP3 pathway through the control of PAX3.

The PAX3 protein recognizes the MITF promoter and prevents its transactivation by MITF, which is involved in melanocyte differentiation; these findings suggest that PAX3 directly regulates MITF [31,32]. In the current study, PAX3 and MITF levels decreased upon MC extract administration, suggesting that the decrease in MITF was induced by a decrease in PAX3. MITF target genes play important roles in melanoma survival, including inducing an increase in the expression of the antiapoptotic gene Bcl2 [33], cell cycle-related gene CDK2 [34], cell motility-related gene c-Met [35], and pigment production/secretion-related gene RAB27A [36]. In the current study, melanoma cell treatment increased the expression of MITF and its downstream genes CDK2, c-Met, Bcl2, and RAB27A. However, MC extract administration decreased the expression of these tumor survival genes; this finding suggests that the MITF pathway is involved in the suppression of melanoma proliferation and invasion by the MC extract. MC extract administration induced the amelioration of melanoma by controlling the PAX3/PTEM/PIP3/Akt/mTORC1 and PAX3/MITF/CDK2·c-Met·Bcl2·RAB27A signaling pathways, which are involved in melanoma proliferation and invasion (Figure 6). However, the mechanism underlying the reduction in PAX3 expression by the MC extract remains unclear and needs to be explored in future studies.

## 4. Materials and Methods

### 4.1. MC Extract Treatment

The MC lyophilizate was provided by ChromaDex, Inc. (Irvine, CA, USA). Approximately 20 volumes of 80% (*v*/*v*) ethanol were added to the dried MC powder samples purchased, and extraction was performed by stirring the mixture overnight at 4 °C. In addition, to obtain a large amount of extract, 20 mL of 80% ethanol was added to 1 g of dried MC powder, and an extract was finally obtained from 10 g of dried powder. The insoluble materials were removed by centrifugation and suction filtration, following which the mixture was concentrated in an evaporator and lyophilized to obtain an ethanol extract [37]. The freeze-dried crude product was orally administered at 50 mg/kg (dissolved in 0.1% DMSO, 200 μL/animal) to the mice thrice a week for 2 weeks. The dosage was determined based on the papers by Kobori et al. [37] and Baek et al. [14] on the effect on cancer, and the maximum effect on melanoma was examined by varying the dosage at 10, 20, 50, 100, and 200 mg/kg. As a result, the maximum effect on melanoma was observed at 50 mg/kg or higher, so 50 mg/kg, which is the minimum concentration with the maximum effect, was adopted in this study. The control group was administered only DMSO [14]. The extracted MC was analyzed for the amount of active ingredients, and the results are shown in Supplementary Table S1.

### 4.2. Animals and Experimental Design

Allotransplantation studies were performed using 15 adult specific pathogen-free (SPF) male 8 week-old C57BL/6j mice weighing 22–27 g (SLC, Hamamatsu, Shizuoka, Japan). In addition, only males were used in this study because females may be affected by hormones due to the sexual cycle. The mice were housed in individual cages in an air-conditioned room at 23 ± 1 °C under SPF conditions with a 12 h light:dark cycle (lights were switched on at 08:00). The animals had ad libitum access to food and water.

The B16 mouse melanoma cell lines established from these tumors were used at passages 5–15 (Japanese Collection of Research Bioresources Cell Bank, Osaka, Japan). All the cell lines were cultured in Eagle’s minimal essential medium (Sigma-Aldrich, Darmstadt, Hesel, Germany) supplemented with a total of 10% serum and l-glutamine. In addition, 5 mL of Penicillin-Streptomycin Mixed Solution (stabilized, nacalai tesqus, tyukyou-ku, Kyoto, Japan) (Penicillin 10,000 units/mL, Streptomycin 10,000 μg/mL) was added as an antibiotic to 500 mL of medium. The cells were routinely tested to ensure that they were free of mycoplasma (Mycoplasma detection kit (Minerva Biolabs GmbH, Skillman, NJ, USA)). For tumor injection, subconfluent monolayers were harvested after treatment with 1 mM 0.25% trypsin–0.02% ethylenediaminetetraacetic acid (Sigma-Aldrich). The trypsinized cells were washed and resuspended in phosphate-buffered saline (PBS; Ca^2+^, Mg^2+^-free phosphate-buffered saline; Sigma-Aldrich).

The animals were randomly assigned to one of three study groups (n = 5/group): control, B16 melanoma injection, or B16 melanoma + MC extract administration group. The group administered the MC extract alone did not differ from the control group in the preliminary test; therefore, we decided to perform this experiment with three groups from the viewpoint of animal welfare (Figure S1). B16 melanoma cells were intravenously injected into the tail vein (1 × 10^5^ cells/mouse) using a Terumo syringe (1 mL) and needle (27G × 3/4” (0.40 × 19 mm)) (TERUMO, Tokyo, Japan). The mice were examined for tumor metastasis 2 weeks after injection. This study was approved by the Suzuka University of Medical Sciences Animal Experimentation Ethics Committee on 25 September, 2019, and was performed in strict accordance with the recommendations of the Guide for the Care and Use of Laboratory Animals at the Suzuka University of Medical Sciences (Approval No: 34). All surgeries were performed under pentobarbital anesthesia, and efforts were made to minimize animal suffering.

### 4.3. Preparation and Staining of Mouse Lung Samples

Lung samples were obtained 2 weeks after the start of the experiment. The lung specimens were fixed in phosphate-buffered paraformaldehyde (4%), embedded in frozen Tissue Tek (optimal cutting temperature compound; Sakura Finetek, Tokyo, Japan), and cut into 5 μm sections. 

To assess the expression of dopa-positive melanocytes, the specimens were incubated in PBS containing 0.1% L-dopa (Sigma-Aldrich) for 3 h at 37 °C; they were then washed with PBS and examined microscopically [38]. Lung specimens were stained using antibodies for immunohistological analysis, according to a previously published method [39]. The following primary antibodies were used for the lung specimens: rabbit polyclonal anti-paired box gene 3 (PAX3; 1:100; Proteintech Group, Rosemont, IL, USA), rabbit polyclonal anti-PTEN (1:100; GeneTex, Inc., Irvine, CA, USA), rabbit polyclonal anti-Akt (1:100; Abcam, Cambridge, MA, USA), mouse monoclonal anti-mTOR (1:100; Proteintech Group), rabbit polyclonal anti-raptor (1:100; Proteintech Group), and rabbit polyclonal anti-rpS6, and rabbit monoclonal anti-S6K1 (1:100; Abcam) primary antibodies. The samples were then washed and incubated with fluorescein isothiocyanate-conjugated anti-mouse and anti-rabbit secondary antibodies (1:30; Dako Cytomation, Glostrup, Denmark).

The PAX3, PTEN, Akt, mTOR, raptor, rpS6, and S6K1 expression levels were immunohistochemically evaluated by using fluorescence microscopy. The gene expression was calculated from five random visual fields with a constant area by using the ImageJ software ver. 1.53 (National Institutes of Health, Bethesda, MD, USA). Briefly, the original files were converted to monochrome 8-bit files. Next, the luminous intensity threshold was voluntarily established. Areas above the threshold were measured for each sample. These areas were defined as “intensity” in this study.

### 4.4. Western Blotting Analysis of Mouse Lung Samples

Lung samples were homogenized in lysis buffer (Kurabo Industries, Osaka, Japan) and centrifuged to obtain supernatants. Western blotting was performed as previously described [40]. After electrophoresis, the membranes were incubated with primary antibodies against MITF (1:1000; Proteintech Group), CDK2 (1:1000; Abcam), c-Met (1:1000; Abcam), Bcl2 (1:1000; Bioss Inc., Woburn, MA, USA), RAB27A (1:1000; Proteintech Group), and β-actin (1:5000; Sigma-Aldrich, St. Louis, MO, USA) for 1 h at room temperature. β-actin was used as the loading control. The membranes were then washed and incubated with horseradish peroxidase-conjugated secondary antibodies (Novex, Frederick, MD, USA). Immune complexes were detected using the ImmunoStar Zeta reagent (Wako Pure Chemical Industries, Osaka, Japan), and images were acquired using the Multi Gauge software ver. 3.0 (Fujifilm, Greenwood, SC, USA).

### 4.5. Measurement of PIP3, PI3K, Ki67, and Cyclin D Levels in Mouse Lungs

Lung samples were obtained on the final day of the experiment; they were isolated and homogenized in a lysis buffer (Kurabo). The tissue extracts were centrifuged at 10,000 rpm (Tomy MX-201; Tomy Digital Biology Co., Ltd., Nerima-ku, Tokyo, Japan), and the supernatants were collected and analyzed. The PIP3, PI3K, cyclin D, and Ki67 levels were measured using commercially available enzyme-linked immunosorbent assay (ELISA) kits (PIP3 and PI3K: MyBioSource, San Diego, CA, USA; cyclin D1: Novus, Centennial, CO, USA; Ki67: Wuhan Fine Biotech, Wuhan, China), according to the manufacturers’ instructions. Optical density was measured using a microplate reader (Molecular Devices, Sunnyvale, CA, USA).

### 4.6. Statistical Analysis

All the data were presented in terms of mean ± SD values. Microsoft Excel 2010 (Microsoft Corp., Redmond, WA, USA) was used to analyze the statistical significance of the data, along with one-way ANOVA, followed by Tukey’s post-hoc test by using SPSS v.20 (SPSS Inc., Chicago, IL, USA). Results with *p*-values < 0.05 (*) and 0.01 (**) were considered statistically significant.

## 5. Conclusions

This study demonstrated that the administration of MC extract improved upon the invasion and proliferation of melanoma. From now on, it is necessary to identify the active components of the MC extract. Furthermore, this study was conducted on mice, and clinical trials on humans are required. Although many tests are still required, if the MC extract is effective against human melanoma, it may help provide inexpensive, highly useful, and safe therapeutic and preventive drugs, and is considered to be an important research in the modern world where the increase in cancer patients is a problem.

## Data Availability

The data presented in this study are available upon request from the corresponding author.

## Supplementary Materials

The following supporting information can be downloaded at: https://www.mdpi.com/article/10.3390/ijms252312800/s1.
