# Supplementary material for: Momordica charantia Extract Ameliorates Melanoma Cell Proliferation and Invasion into Mouse Lungs by Suppressing PAX3 Expression
Source: Int J Mol Sci. 2024 Nov 28;25(23):12800. doi: 10.3390/ijms252312800 (PMC11640897; doi:10.3390/ijms252312800)
Supplement: Supplementary file 1 [file ijms-25-12800-s001.zip › ijms-3325691-supplementary.pptx]

## Slide 1
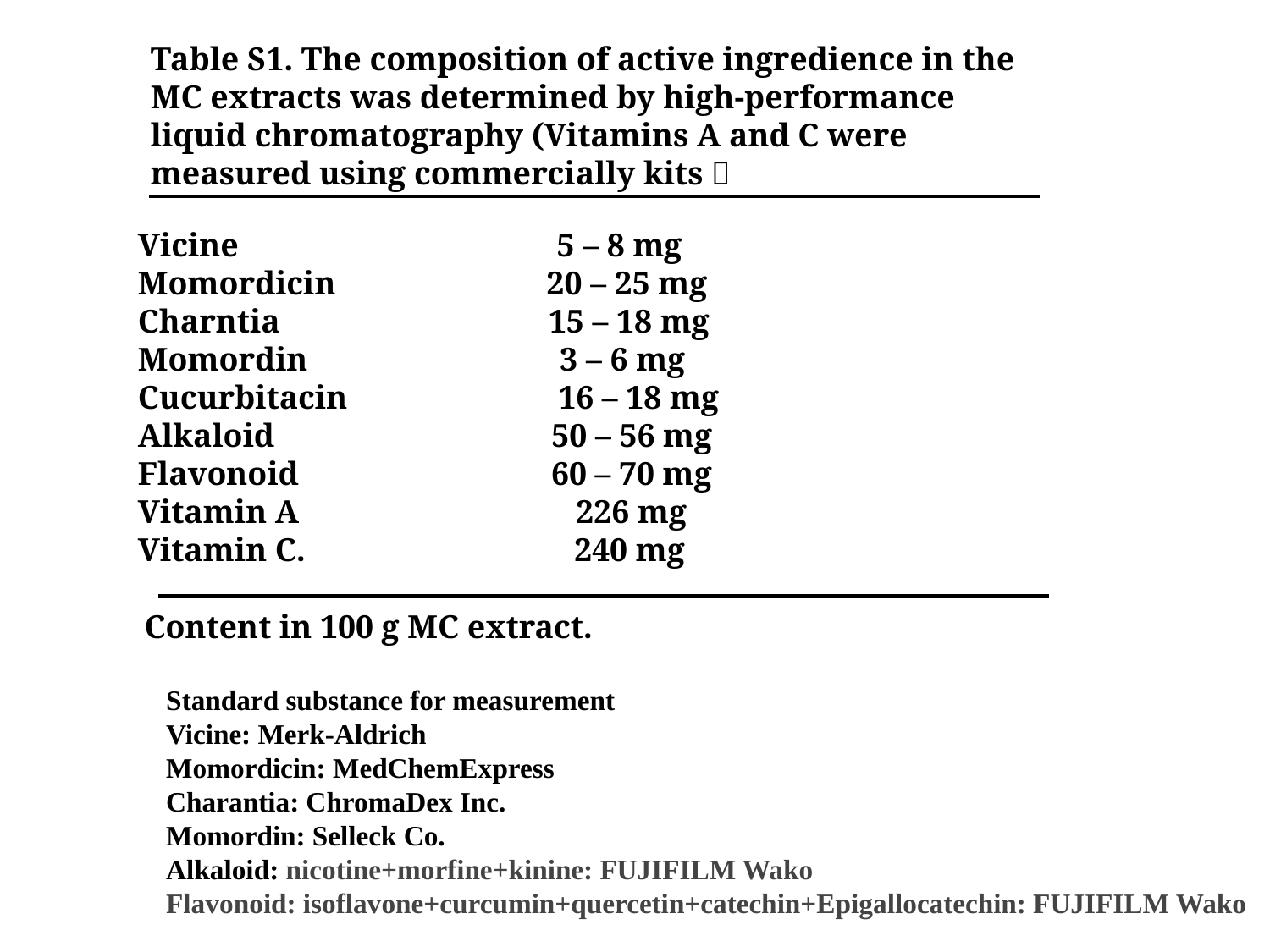

Table S1. The composition of active ingredience in the MC extracts was determined by high-performance liquid chromatography (Vitamins A and C were measured using commercially kits）
Vicine 　　 5 – 8 mg
Momordicin 　　 20 – 25 mg
Charntia 　　 15 – 18 mg
Momordin 　　 3 – 6 mg
Cucurbitacin 　　 16 – 18 mg
Alkaloid 　　 50 – 56 mg
Flavonoid 　　 60 – 70 mg
Vitamin A 　　 226 mg
Vitamin C. 　　 240 mg
Content in 100 g MC extract.
Standard substance for measurement
Vicine: Merk-Aldrich
Momordicin: MedChemExpress
Charantia: ChromaDex Inc.
Momordin: Selleck Co.
Alkaloid: nicotine+morfine+kinine: FUJIFILM Wako
Flavonoid: isoflavone+curcumin+quercetin+catechin+Epigallocatechin: FUJIFILM Wako

## Slide 2
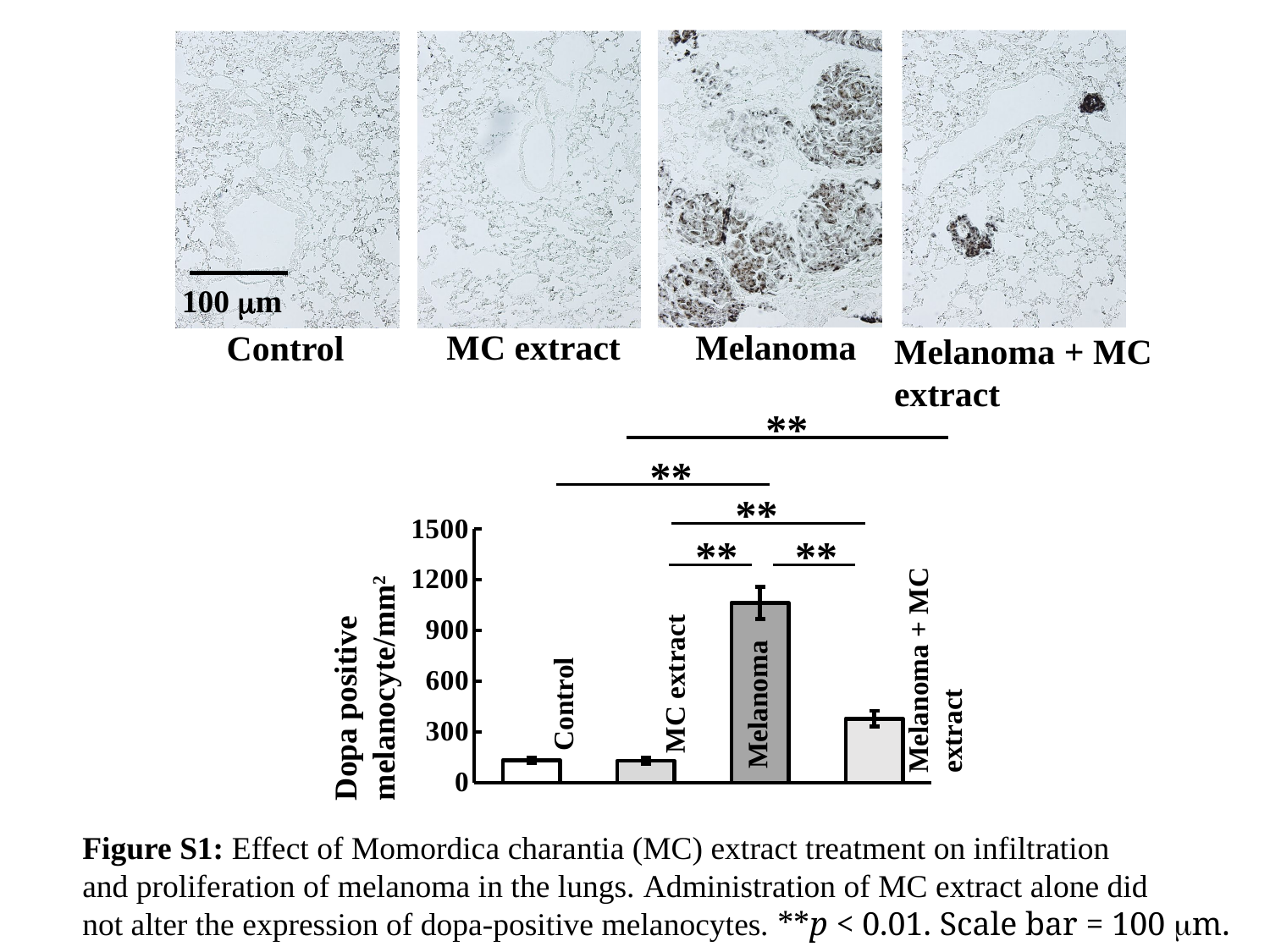

100 mm
MC extract
Melanoma
Control
Melanoma + MC extract
**
**
**
### Chart
| Category | |
|---|---|**
**
Dopa positive melanocyte/mm2
Melanoma + MC extract
MC extract
Control
Melanoma
Figure S1: Effect of Momordica charantia (MC) extract treatment on infiltration
and proliferation of melanoma in the lungs. Administration of MC extract alone did
not alter the expression of dopa-positive melanocytes. **p < 0.01. Scale bar = 100 mm.
